# Antiviral role of IFITM3 in prototype foamy virus infection

**DOI:** 10.1186/s12985-022-01931-x

**Published:** 2022-11-22

**Authors:** Zhaohuan Wang, Xiaopeng Tuo, Junshi Zhang, Keli Chai, Juan Tan, Wentao Qiao

**Affiliations:** 1 Key Laboratory of Molecular Microbiology and Technology, Ministry of Education, College of Life Sciences, Nankai University, Tianjin, 300071 China; 2 Present Address: Merck Sharp & Dohme Corp, Building 21, Rongda Road, Chaoyang District, Beijing, 1000102 People’s Republic of China; 3 Present Address: Department of Hematology, Oncology Centrer, Tianjin People’s Hospital, No. 190 Jieyuan Road, Hongqiao District, Tianjin, 300121 People’s Republic of China; 4 Present Address: Jiangsu Center for the Collaboration and Innovation of Cancer Biotherapy, Cancer Institute, Xuzhou Medical University, Xuzhou, 221002 Jiangsu China

**Keywords:** IFITM3, Prototype foamy virus, Entry, Envelope

## Abstract

**Background:**

Foamy viruses (FVs) are retroviruses with unique replication strategies that cause lifelong latent infections in their hosts. FVs can also produce foam-like cytopathic effects in vitro. However, the effect of host cytokines on FV replication requires further investigation. Although interferon induced transmembrane (IFITMs) proteins have become the focus of antiviral immune response research due to their broad-spectrum antiviral ability, it remains unclear whether IFITMs can affect FV replication.

**Method:**

In this study, the PFV virus titer was characterized by measuring luciferase activity after co-incubation of PFVL cell lines with the cell culture supernatants (cell-free PFV) or the cells transfected with pcPFV plasmid/infected with PFV (cell-associated PFV). The foam-like cytopathic effects of PFV infected cells was observed to reflect the virus replication. The total RNA of PFV infected cells was extracted, and the viral genome was quantified by Quantitative reverse transcription PCR to detect the PFV entry into target cells.

**Results:**

In the present study, we demonstrated that IFITM1-3 overexpression inhibited prototype foamy virus (PFV) replication. In addition, an IFITM3 knockdown by small interfering RNA increased PFV replication. We further demonstrated that IFITM3 inhibited PFV entry into host cells. Moreover, IFITM3 also reduced the number of PFV envelope proteins, which was related to IFITM3 promoted envelope degradation through the lysosomal pathway.

**Conclusions:**

Taken together, these results demonstrate that IFITM3 inhibits PFV replication by inhibiting PFV entry into target cells and reducing the number of PFV envelope.

**Supplementary Information:**

The online version contains supplementary material available at 10.1186/s12985-022-01931-x.

## Introduction

Foamy viruses (FVs) belong to the *Spumaretrovirinae* subfamily of *Retroviridae*, and their replication strategy is intermediate between the *Hepadnaviridae* and *Retroviridae*. [1, 2]. Envelope proteins are essential for FV replication and the budding of FVs is strictly envelope glycoprotein-dependent since their Gag proteins lack membrane-targeting signals and must interact with envelope proteins for membrane-targeting ability [3, 4]. Similar to the hepatitis B virus S protein, FVs release Env-only subviral particles [3]. FVs have a wide range of hosts and infect primates, felines, bovines, and equines [5–8]; however, there have been few studies on the effects of cellular factors on FV replication.

The innate immune response plays an important role in antiviral therapy. Interferon (IFN)-induced transmembrane proteins (IFITMs) are activated by the interferon stimulating genes (ISGs), which have recently become an area of research focus on the antiviral immune response due to their broad-spectrum antiviral effects [9]. At present, there are five genes encoding IFITM proteins in humans, including *IFITM1*, *IFITM2*, *IFITM3*, *IFITM5*, and *IFITM10* [10]. IFITM1-3 proteins can be significantly upregulated by IFNs and are ubiquitously expressed in human tissues in the absence of IFN induction; however, IFITM5 and IFITM10 protein expression was not induced by IFN [11]. IFITM1, IFITM2, and IFITM3 play a role in embryonic development, signal transduction, tumorigenesis, and antiviral activities [12, 13]. IFITM5, which is involved in bone mineralization and maturation, is only expressed in osteoblasts, whereas the function of IFITM10 remains unclear [14, 15]. Some other species have been reported to exhibit homologous genes in the IFITM family, suggesting that IFITMs have important and conserved functions [16].

At present, the antiviral spectrum of IFITMs encompasses over 20 viruses from 12 families [15]. These include DNA viruses, enveloped RNA viruses and non-enveloped RNA viruses [17–19]. There are several viruses with severe pathogenicity in humans that are inhibited by IFITMs, including human immunodeficiency virus (HIV) [20], Ebola virus (EBOV) [21], influenza A virus (IAV) [22, 23], Zika Virus (ZIKV) [24] and severe acute respiratory syndrome coronavirus (SARS-CoV) [25]. Among those with antiviral activity, IFITM3 is the best characterized [26]. Moreover, there are some viruses that are not inhibited by IFITMs, including murine leukemia virus (MLV) [27], adeno-associated virus (AAV) [28], lymphocytic choroid plexus bacterial meningitis (LCMV) [29], and arenavirus (LASV) [15]. Further research is required to clarify the molecular mechanisms underlying IFITM antiviral activity and why some viruses can escape IFITM inhibition.

At present, the comprehensive antiviral mechanism of IFITM primarily includes IFITM-mediated inhibition of viral entry by inhibiting viral fusion to the plasma membranes and lysosomal or endosomal membranes, rather than relying on specific recognition of viral components to restrict virus entry [9, 26]. IFITM proteins can also regulate the endosomal or lysosomal pH [30]. The conformation of some viral envelope proteins (e.g., hemagglutinin) changes at a low endosomal pH, mediating hemifusion of viral and endosomal membranes [31]. In addition, IFITM3 can inhibit the replication of some non-enveloped viruses by regulating the function of late endosomes [18]. IFITMs have also been demonstrated to reduce the infectivity of some newly generated viruses. For example, IFITM proteins can be colocalized with HIV-1 Env and Gag and become part of the nascent generated virus particles, inhibiting virion entry into new host cells [20]. Recently, IFITM proteins have been found to inhibit HIV-1 protein synthesis and thus limit viral infection [32]. Prototype foamy virus (PFV) is a type of FV. However, whether the replication of PFV, an enveloped virus that entry target cells in a pH-dependent manner [33], is affected by IFITMs remains unclear. In this study, we demonstrated that IFITM1-3 inhibits PFV replication by inhibiting PFV entry and reducing the number of PFV envelope protein.

## Materials and methods

### Plasmid constructs

Full-length infectious clone of PFV (pcPFV) was kindly provided by Maxine L. Linial (Division of Basic Sciences, Fred Hutchinson Cancer Research Center, Seattle, WA, USA) [34]. The pCMV-Tag2B-Tas plasmid was constructed by inserting the 9434 to 10,336 nucleotide region containing *orf1* into pCMV-Tag2B. HA-tagged IFITM1-3 eukaryotic expression plasmids were constructed by inserting the cDNA of human *IFITM1*, *IFITM2*, and *IFITM3* into pCMV-3HA vectors (Clontech, Mountain View, CA, USA). Insert the cDNA encoding human *IFITM3* into pQCXIP-Flag vector to construct a pQCXIP-Flag-IFITM3 plasmid. The PFV envelope encoding sequence was inserted into pCMV-3HA or pCE-puro-3×FLAG to construct the pCMV-3HA-Env or pCE-puro-3×Flag-Env plasmid. The pEGFP-C3 vector was purchased from BD Biosciences (Clontech, Mountain View, CA, USA). A pair of double-stranded oligonucleotides targeting *IFITM3* was inserted into the pSIREN-RetroQ vector (Clontech, Mountain View, CA, USA) to obtain the pSIREN-RetroQ-shIFITM3 plasmid.

### Cell culture and transfection

Human embryonic kidney (HEK293T), PFVL (BHK21-derived indicator cell lines containing the *luciferase* gene initiated by the PFV LTR) [35], HT1080, and HeLa cells were incubated in Dulbecco's modified Eagle's medium (Gibco, Thermo Fisher Scientific, Waltham, MA, USA) containing 10% fetal bovine serum (Gibco) at 37 °C in 5% CO2. Plasmid transfection was performed using polyethyleneimine (PEI, Polysciences, Warrington, PA, USA) reagent according to the manufacturer's instructions [36].

### Virus production and infection

The pcPFV plasmid was transfected into HEK239T cells and the cell culture supernatant was collected 48 h after transfection and filtered through a 0.45 µm filter to obtain the PFV virus stock solution, which can be stored at 4℃. PFV viral titers were measured by infection of PFVL cells [37]. Target cells were infected with the virus stock and fresh culture medium was replaced at 8 h post-infection. After 48 h of infection, 1/20 of the infected cells or 500 µL of cell culture supernatant were co-incubated with PFVL cells to test the infectivity of the virus produced by replication in target cells. The levels of PFV protein expression in the infected cells were analyzed by Western blotting.

### Generation of knockdown cell lines

We used small hairpin RNA (shRNA) to screen IFITM3 knockdown cell lines. The target sequences of IFITM3 shRNA and control shRNA were 5′-TCCCACGTACTCCAACTTCCA-3′ and 5′-GAAGTAAGCGATATACATA-3′, respectively. HEK293T cells were co-transfected with 1 µg pSIREN-RetroQ-shIFITM3 or pSIREN-RetroQ-shControl plasmids, 1 µg pMVL-Gag/Pol, and 0.5 µg pVSV-G. HT1080 cells were infected with the cell culture supernatants at 48 h after transfection. At 48 h post-infection, knockdown HT1080 cells were selected by medium containing puromycin (2 μg/mL). The following primer sequences were used to detect IFITMs mRNA levels in HT1080-shIFITM3 cells by qPCR: IFITM1-up: 5′-ATCCTGTTACTGGTATTCGG-3′; IFITM1-low: 5′-TATAAACTGCTGTATCTAGG-3′; IFITM2-up: 5′-GTTGGTCGTCCAGGCCCAGC-3′; IFITM2-low: 5′-CTGTGGGGACAGGGCGAGGA-3′; IFITM3-up: 5′-GCTGATCTTCCAGGCCTATG-3′; IFITM3-low: 5′-GATACAGGACTCGGCTCCGG-3′.

### Luciferase reporter assay

Following a co-incubation with cell culture supernatants (containing virus particles) or PFV-infected cells, luciferase levels of PFVL cells were determined using a luciferase reporter assay system (Promega, Madison, WI, USA). The corresponding results were the average of three independent experiments.

### Alu-PCR

HT1080 cells transfected with IFITM3 expression plasmid or control vector were infected with the PFV virus stock, and the total DNA of infected cells was extracted using a DNeasy Blood and Tissue Kit (Qiagen, Duesseldorf, Germany) 30 h post-infection. Cells treated with 10 µM of the integrase inhibitor raltegravir or 10 µM of the reverse transcriptase inhibitor AZT were used as controls, the degree of PFV genome integration was detected by semi-quantitative PCR and real-time PCR using Alu-PCR primers.

The extracted total DNA (100 ng) was used as template, and 10 µM of Alu1, Alu2 and SpA primers were added for PCR. PCR conditions were 95 °C for 5 min for 1 cycle; 95 °C for 30 s, 55 °C for 30 s, and 68 °C for 3 min, for 22 cycles; 68 °C for 7 min for 1 cycle. Next 2 µL PCR products were used as the template, and 10 µM Lambda and Nested-R primers were added for the second PCR. PCR conditions were: 95 °C for 5 min for 1 cycle; 95 °C for 30 s, 55 °C for 30 s, and 68 °C for 30 s, for 22 cycles; 68 °C for 7 min for 1 cycle. Using the extracted total DNA as a template, the *gapdh* gene was amplified as a control. PCR conditions were 95 °C for 5 min for 1 cycle, 95 °C for 30 s, 60 °C for 30 s, and 68 °C for 30 s, for 22 cycles, and 68 °C for 7 min for 1 cycle. The following primer sequences were used: SpA: 5′-ATGCCACGTAAGCGAAACTTAGTATAATCATTTCCGCTTTCG-3′; GAPDH-up: 5′-AACAGCGACACCCACTCCTC-3′, GAPDH-low: 5′-CATACCAGGAAATGAGCTTGACAA-3′; Lambda: 5′-ATGCCACGTAAGCGAAACT-3′; NestedR: 5′-GAAACTAGGGAAAACTAGG-3′; Alu1: 5′-TCCCAGCTACTGGGGAGGCTGAGG-3′, Alu2: 5′-GCCTCCCAAAGTGCTGGGATTACAG-3′.

### Western blotting

The cell lysates were placed on ice for 30 min after adding lysis buffer (1% NP-40, 3% Glycerol, 2 mM EDTA, 50 mM Tris, 150 mM NaCl) to the cell samples to be tested. The proteins were subjected to sodium dodecyl sulfate polyacrylamide gel electrophoresis (SDS-PAGE) and subsequently transferred to polyvinylidene difluoride (PVDF) membranes (GE Healthcare, Cincinnati, OH, USA). The PVDF membrane was blocked with 5% nonfat milk for 45 min, after which the PVDF membrane was incubated with primary antibodies for 1.5 h and incubated with peroxidase-conjugated secondary antibodies for 45 min. Immunoreactive protein signals were detected by chemiluminescence (Merck Millipore, Darmstadt, Germany).

### Antibodies

The following antibodies were used in the protein detection analysis in this study: polyclonal rabbit anti-IFITM3 (1:2000; cat. no. 11714-1-AP, Proteintech, Chicago, IL, USA), monoclonal mouse anti-HA (1:3000; cat. no. H3663, Sigma-Aldrich, St. Louis, MO, USA), monoclonal mouse anti-GFP (1:2000; cat. no. sc-9996, Santa Cruz Biotechnology, Dallas, TX, USA), monoclonal mouse anti-Tubulin (1:5000; cat. no. sc-32293, Santa Cruz), monoclonal mouse anti-GAPDH (1:5000; cat. no. sc-47724, Santa Cruz Biotechnology, Dallas, TX, USA), horseradish peroxidase (HRP)-conjugated goat anti-mouse IgG (1:5000; cat. no. sc-2005, Santa Cruz), and HRP-conjugated goat anti-rabbit IgG (1:5000; cat. no. sc-2004, Santa Cruz), and monoclonal mouse anti-Flag (1:5000; cat. no. F1804, Sigma-Aldrich) antibodies. We use purified Tas or PFV Gag (180–433 aa) proteins as immunogens to immunize BALB/c mice to obtain antibodies against the corresponding proteins. Since part of the Bet protein overlaps with the 88 amino acids at the N terminus of the Tas protein, the Bet protein can be detected using anti-serum prepared with the Tas protein as an immunogen.

### Virus entry assay

HT1080 cells were infected with a PFV stock solution and incubated at 4℃ for 1 h to ensure virus attachment to the cell membrane surface but not entry into cells. The cells were washed with PBS to remove any unattached virus, and the cells were cultured at 37℃ after replacing the culture medium. After 0 h, 2 h, 4 h 6 h and 8 h, the cells were harvested to extract the total RNA from the infected cells. The number of viral genomes in the cells was detected by RT-qPCR to indicate the level of viral entry. The amount of viral genome was indicated by the level of Gag gene expression. The following primers were used for RT-qPCR: Gag forward (5′-AATAGCGGGCGGGGACGACA-3′), Gag reverse (5′-ATTGCCACGCACCCCAGAGC-3′); GAPDH forward (5′-AACAGCGACACCCACTCCTC-3′), GAPDH reverse (5′-CATACCAGGAAATGAGCTTGACAA-3′).

### Statistical analysis

The data were presented as the mean ± standard deviations (SD) of the results of three independent experiments. The data were analyzed using GraphPad Prism Version 8.0 (GraphPad software Inc., San Diego, CA, USA). When the *P* value was greater than 0.05, the difference was not significant (ns). A *P* value less than 0.05 indicated a statistically significant difference (*P < 0.05; **P < 0.01; ***P < 0.001; ****P < 0.0001).

## Results

### IFITM3 inhibits PFV entry into HT1080 cells

**Fig. 1:**
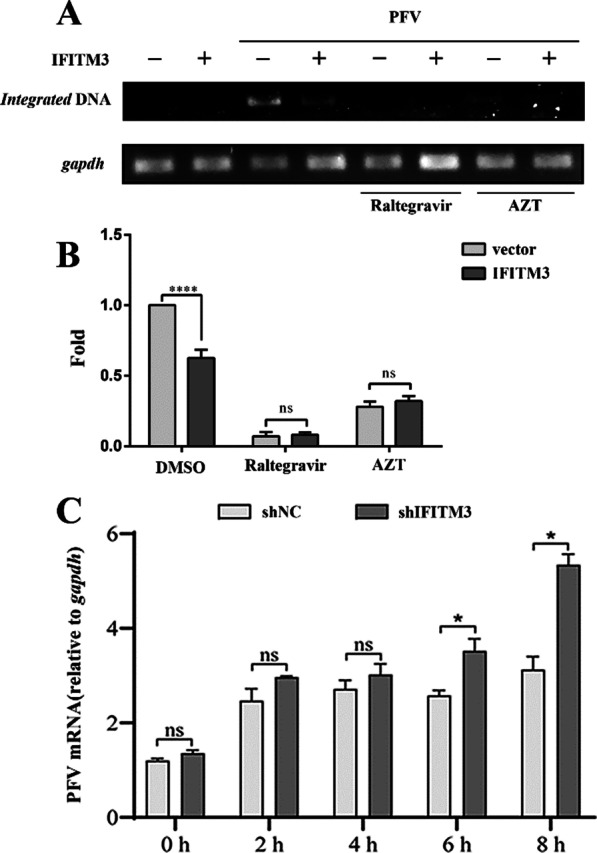
IFITM3 inhibits PFV entry into HT1080 cells. HT1080 cells were infected with the PFV virus stock solution (2 MOI) 24 h after transfection of IFITM3 expression plasmid or control vector, and total DNA was extracted 30 h after infection. The semiquantitative PCR (**A**) and real-time PCR (**B**) results indicate the level of proviral DNA integration. **C** The levels of PFV entry were evaluated as described in the Materials and Methods. The amount of PFV *Gag* gene relative to *gapdh* indicates the level of viral mRNA. Data are representative of three independent experiments. Values were statistically evaluated using a two-way ANOVA. *P < 0.05; **P < 0.01; ***P < 0.001; ****P < 0.0001. Error bars represent the standard deviations

Studies have shown that IFITM proteins can broadly inhibit the entry of a variety of viruses into target cells [21, 26, 38, 39]. Whether IFITM3 affects the entry of PFV has not been reported. To clarify whether IFITM3 inhibits early step in the PFV life cycle, we first examined whether IFITM3 affected the PFV genome integration. The integration of the PFV genome into PFV-infected cells was detected by Alu-PCR. Based on the principle of Alu-PCR, the integrated PFV genome was detected using semi-quantitative PCR (Fig. 1A) and real-time PCR (Fig. 1B), respectively. As shown in the Fig. 1A, B, the GAPDH band intensity was similar in all experimental groups. PFV genome integration was inhibited following treatment with raltegravir (an integrase inhibitor) or AZT (a reverse transcriptase inhibitor). In addition, the degree of integration of PFV in the IFITM3 overexpression group under the treatment of these two inhibitors was the same as that of the respective control group. IFITM3 overexpression inhibited PFV genome integration when without inhibitor treatment. These results suggested that IFITM3 might influence PFV integration and some steps prior to integration during replication.

Therefore, we next explored whether IFITM3 affects PFV entry into target cells by first infecting HT1080-shNC or HT1080-shIFITM3 cells with PFV virus stock solution. The degree of viral entry into the cells was measured at various time points post-infection. As shown in Fig. 1C, after a 6 h culture at 37℃, the level of PFV entry in the IFITM3 knockdown cells was higher than that in the control group, demonstrating that IFITM3 inhibited PFV entry into HT1080 cells.

### An IFITM3 knockdown promotes PFV replication

**Fig. 2:**
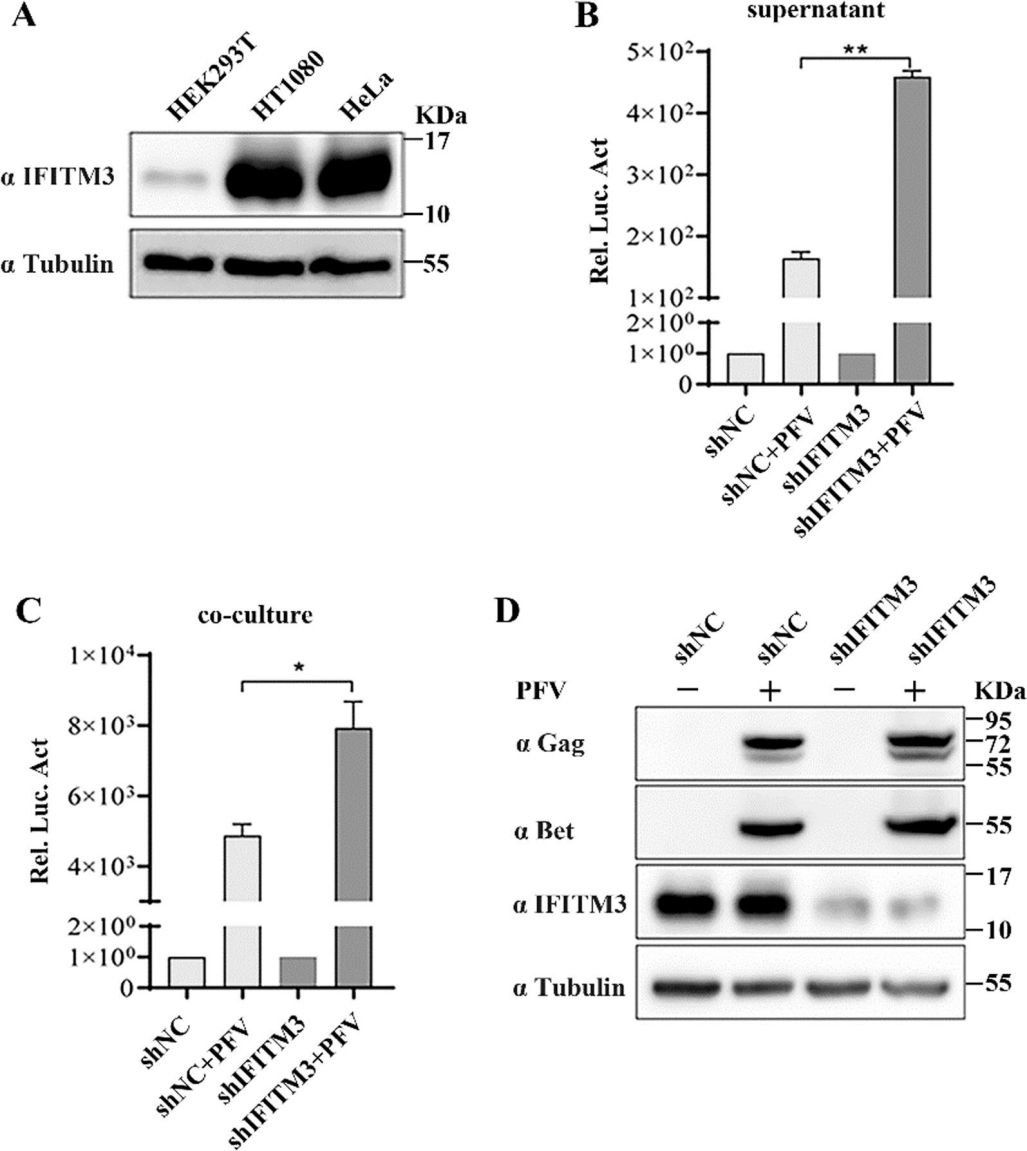
IFITM3 knockdown promotes PFV replication. **A** Levels of IFITM3 protein expression in HT1080, HEK293T, and HeLa cells. **B** and **C** Infection of HT1080-shNC or HT1080-shIFITM3 cells with a PFV virus stock solution (0.49 MOI). At 48 h post-infection, the PFVL indicator cell lines were co-incubated with cell culture supernatants (1/2 µL) or HT1080 cells (1/20). The level of luciferase activity was measured at 48 h after co-incubation (values were statistically evaluated using t tests. compared with shNC + PFV group, *P < 0.05; **P < 0.01). Error bars represent the standard deviations. **D** The level of PFV proteins in HT1080 cells. Data are representative of three independent experiments

To further clarify the inhibitory effect of IFITM3 on PFV replication, we examined the effect of an IFITM3 knockdown on PFV replication. The level of endogenous IFITM3 in HT1080 and HeLa cells was similar, which was higher than that in HEK293T cells (Fig. 2A). We screened HT1080 cell lines with an endogenous IFITM3 knockdown, due to the high sequence similarity of IFITM1, IFITM2 and IFITM3, to ensure that IFITM3 knockdown was specific, we detected the mRNA levels of IFITM1, 2 and 3 in HT1080-shIFITM3 cell line. As shown in the Additional file 1: Fig. S1, the knockdown of IFITM3 in HT1080-shIFITM3 cells was specific, IFITM3 knockdown did not affect the mRNA levels of IFITM1 and IFITM2. IFITM3 knockdown HT1080 cells were infected with a PFV viral stock solution. At 48 h post-infection, the PFVL cell lines were incubated with the cell culture supernatant or infected HT1080 cells. The levels of viral protein in the HT1080 cells were also detected. After a 48 h incubation, the luciferase activity in PFVL cells was measured. An IFITM3 knockdown was found to enhance PFV replication in HT1080 cells (Fig. 2B–D). This finding indicated that endogenous IFITM3 inhibited PFV replication, which was consistent with the results of Fig. 1.

### IFITM3 inhibits PFV passaging in HT1080 cells

**Fig. 3:**
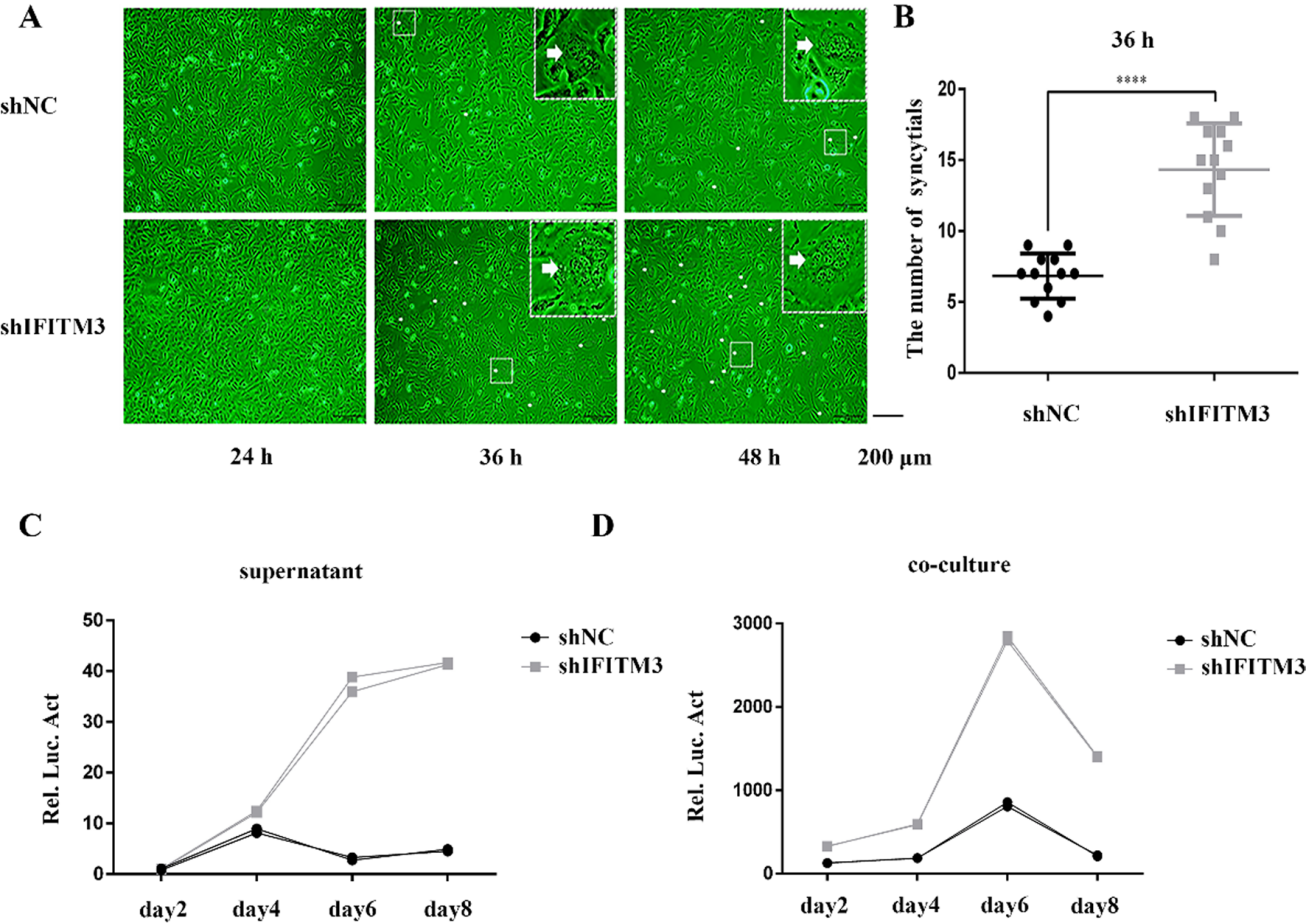
Knockdown of IFITM3 promotes the passage of PFV. **A** HT1080-shNC or HT1080-shIFITM3 cells were infected with the PFV virus stock (0.49 MOI). The cellular state and pathological changes were observed using an electron microscope at different time points following viral infection. The white arrows indicate syncytium. **B** The number of syncytia in multiple random fields 36 h after virus infection in **A** was counted. A total of 12 fields of view were included in three independent experiments. **C**, **D** HT1080-shNC or HT1080-shIFITM3 cells were infected with the PFV virus stock (0.17 MOI). Every 2 days following infection, the PFVL indicator cell lines were co-incubated with cell culture supernatants (1/2 µL) or HT1080 cells (1/20). Infected cells (0.03 × 10^6^) were supplemented with 10 times the number of uninfected shNC or shIFITM3 cells. The measured luciferase level of PFVL after co-incubation indicated the titer of the virus after passaging. Values were statistically evaluated using t tests. *P < 0.05; **P < 0.01; ***P < 0.001; ****P < 0.0001. Data are representative of two (**C**, **D**) or three (**B**) independent experiments

PFV infection of cultured cells induces severe cytopathic effects (CPE) [40]. To more visually reflect the effect of IFITM3 on PFV replication, we infected IFITM3 knockdown or control HT1080 cell lines with a PFV virus stock solution, and observed the state of the cells at different time points following infection. As shown in Fig. 3A, B, both the shNC and shIFITM3 cells showed foam-like cytopathic (syncytium formation) effects to a certain extent at 36 h post-infection, the syncytia of the shNC and shIFITM3 cell lines increased at 48 h post-infection, and the syncytia production of shIFITM3 cell lines at 36 h and 48 h post-infection was stronger than that of shNC cell lines.

We further examined the passage of PFV in shNC or shIFITM3 cell lines. Upon PFV infection of shNC or shIFITM3 HT1080 cell lines, the cell culture supernatant and infected HT1080 cells were harvested every 48 h and incubated with the PFVL indicator cell lines. Luciferase activity was measured after an incubation for two days. A total of 0.03 × 10^6^ infected HT1080 cells were retained and 10 times the number of uninfected shNC or shIFITM3 cells were supplemented. The titer of the viral passage was indicated by the level of luciferase activity as measured every 48 h. As shown in Fig. 3C, D, little difference was observed in the PFV titer between shNC and shIFITM3 cells at the beginning of the passage. However, starting from day 4, the PFV virus titer passage in the shIFITM3 cells was significantly higher than that in the control group. These results further confirmed the inhibition of PFV replication by IFITM3.

### IFITMs overexpression inhibits the late step of PFV replication

**Fig. 4:**
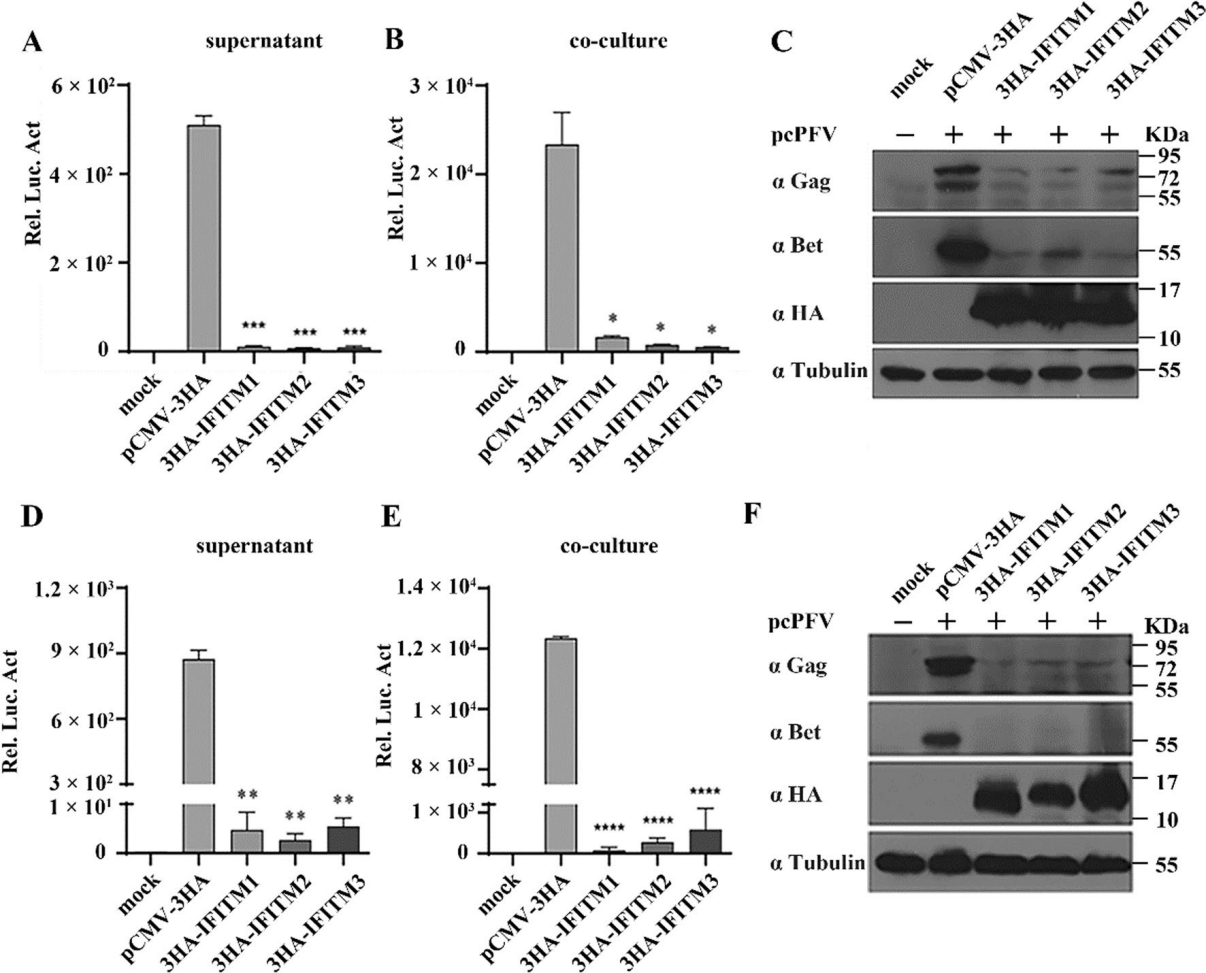
IFITMs overexpression inhibits the late step of PFV replication. **A**, **B** The pcPFV was co-transfected into HEK293T cells with IFITM eukaryotic expression plasmids or control vector, respectively. At 48 h post-transfection, the PFVL indicator cell lines were co-incubated with cell culture supernatants (1/2) or transfected HEK293T (1/20) cells. The level of luciferase was measured 48 h after co-incubation. Values were statistically evaluated using one-way ANOVA. Compared with the pcPFV and control vector co-transfection group: *P < 0.05; **P < 0.01; ***P < 0.001; ****P < 0.0001. Error bars represent the standard deviations. **C** The level of PFV protein expression in transfected HEK293T cells. **D**–**F** We performed the above experiments in HT1080 cells. Data are representative of three independent experiments

The IFITM proteins were reported to inhibit the late step of feline foamy virus (FFV) replication [41]. To investigate whether IFITM proteins have an effect on the late step of PFV replication, pcPFV and IFITM1, IFITM2, or IFITM3 plasmids were cotransfected into HEK293T cells (the effect of IFITMs on PFV entry was excluded). At two days post-transfection, PFVL indicator cell lines were incubated with the cell culture supernatants (cell-free PFV) or the transfected HEK293T cells (cell-associated PFV). Luciferase activity was detected following 48 h incubation to indicate PFV replication. At the same time, the level of viral proteins in the transfected HEK293T were detected. In Fig. 4A, B, IFITM overexpression significantly reduced luciferase activity, indicating decreased cell-free and cell-associated PFV. Correspondingly, the levels of PFV Bet and Gag protein expression were also significantly reduced in transfected HEK293T cells (Fig. 4C). We performed the same experiments in HT1080 cells and obtained similar results (Fig. 4D–F). Taken together, these results indicate that IFITM1, IFITM2 or IFITM3 overexpression inhibited the late step of PFV replication.

### IFITM3 inhibits the amount of PFV envelope protein by degrading it through the lysosomal pathway

**Fig. 5:**
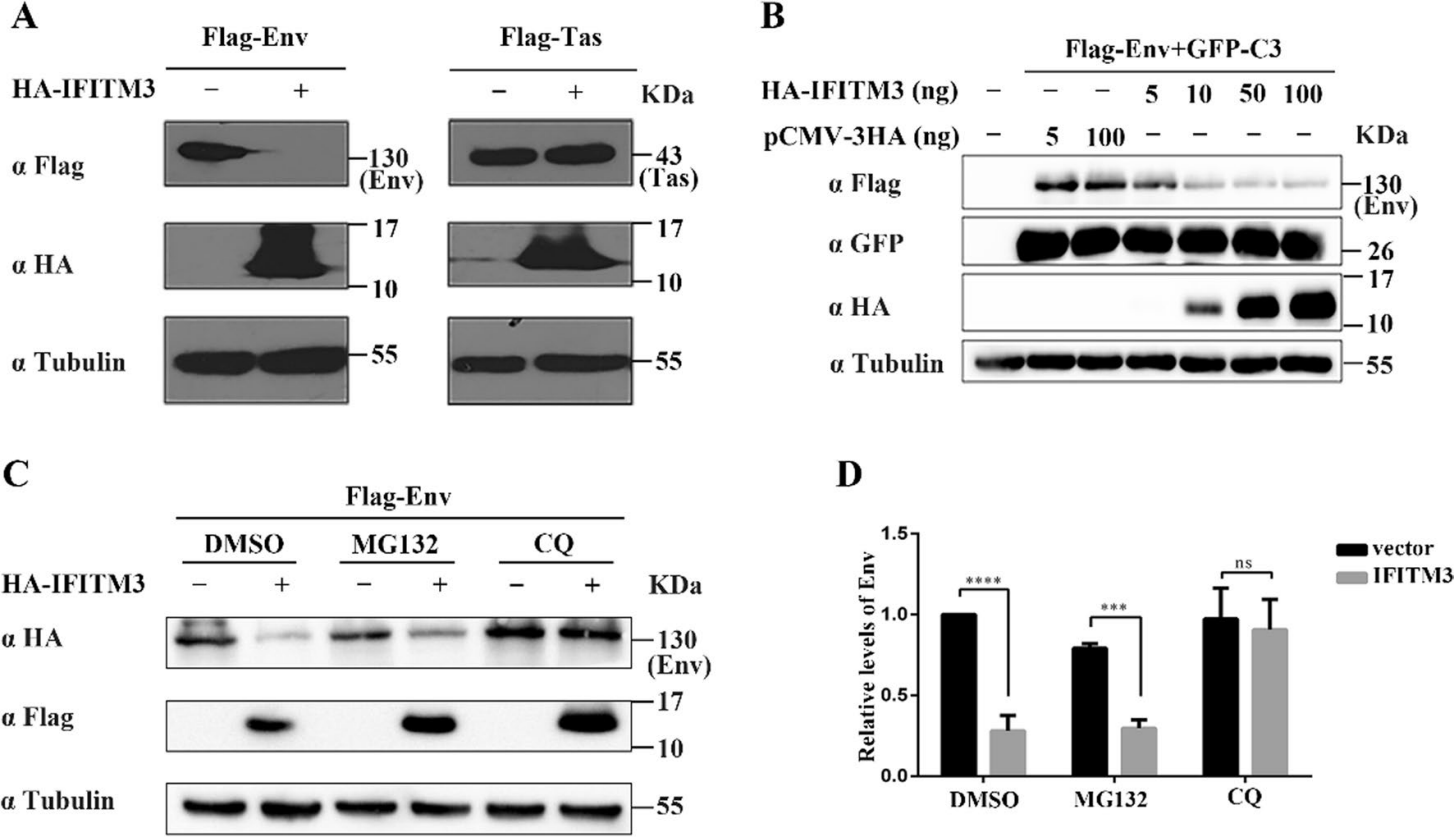
IFITM3 reduces the amount of the PFV envelope protein. **A** HEK293T cells were co-transfected with plasmids encoding for the PFV envelope or Tas protein with HA-IFITM3 or control vector, respectively. The level of intracellular protein expression was detected at 48 h post-transfection. **B** Flag-Env and GFP-C3 plasmids were co-transfected with HA-IFITM3 or control vector in HEK293T cells. The level of protein expression was detected at 48 h post-transfection. **C** HEK293T cells were co-transfected with HA-Env and Flag-IFITM3 or control vector. At 30 h post-transfection, the cells were treated with DMSO (100 µM), MG132 (10 µM), or CQ (100 µM). The level of protein expression was detected at 48 h post-transfection. **D** Quantification of Env protein levels in **C**. Mean values and standard deviations of Env proteins corrected for Tubulin levels (n = 3) are shown. Values were statistically evaluated using a two-way ANOVA. *P < 0.05; **P < 0.01; ***P < 0.001; ****P < 0.0001

The results in Fig. 4 showed that IFITM1, IFITM2 or IFITM3 overexpression inhibited PFV replication in virus-producing cells to similar degrees. Studies have shown that IFITM3 can reduce the abundance of retroviral envelope proteins [42]. Therefore, we detected the effect of IFITM3 on PFV envelope protein expression. IFITM3 reduced the number of PFV envelope protein without affecting the number of the PFV regulatory protein, Tas (Fig. 5A). We then co-transfected the envelope and GFP plasmids with a certain amount of IFITM3. As shown in Fig. 5B, IFITM3 reduced the number of envelope protein without affecting the level of GFP protein expression. This result indicated that the effect of IFITM3 on the PFV envelope protein is specific. The reduction in envelope protein expression by IFITM3 was dose-dependent, and a small amount (10 ng) of IFITM3 was sufficient to significantly reduce the number of the PFV envelope protein. To detect whether the influence of IFITM3 on the number of PFV envelope was related to the degradation, we examined the effect of IFITM3 on envelope expression following treatment with MG132 (a proteasome inhibitor) or Chloroquine (CQ, an inhibitor of lysosomal acidity and function). As shown in Fig. 5C, D, under DMSO or MG132 treatment, IFITM3 could significantly reduce the levels of envelope, whereas IFITM3 only had a minor effect on the number of PFV envelope under CQ treatment. These results suggest that the reduction of envelope by IFITM3 is correlated with degradation through the lysosomal pathway.

## Discussion

At present, IFITMs have been demonstrated to antagonize a variety of viruses, including several serious pathogenic viruses, including human immunodeficiency virus (HIV) [43], Ebola virus (EBOV) [21], and Zika Virus (ZIKV) [24]. In addition, there are some viruses that are able to escape the confinement of IFITMs, such as murine leukemia virus (MLV) [27], adeno-associated virus (AAV) [28] and arenavirus (LASV) [15]; however, the antiviral properties of IFITMs and the reason that some viruses are able to evade inhibition by IFITMs remain unclear. Further research on the effect of IFITMs on diverse viral replication will help clarify the antiviral mechanism of IFITMs.

In this study, we demonstrated that IFITM1-3 could significantly inhibit PFV, and interestingly they inhibited PFV to a similar degree. Previous studies have shown that different IFITMs tend to inhibit the same virus to different degrees. For example, compared with IFITM1 and IFITM2, IFITM3 has a significantly strong effect on influenza A virus (IAV) [23] and IFITM3 can inhibit Zika virus (ZIKV) replication more effectively than IFITM1 [24]. Moreover, the degree of HIV-1 inhibition varies among IFITM members (IFITM3 > IFITM2 > IFITM1) [20], and is related to many factors. Such factors include physiological and biochemical characteristics of IFITMs (e.g., the differences in IFITM localization) and the molecular characteristics of virus replication (e.g., the position of the virus entering the target cell). A similar degree of PFV-mediated inhibition by IFITM1-3 may be related to the unique replication strategy of PFV, the specific mechanism of which requires further study.

IFITM1, IFITM2 and IFITM3 have been reported to inhibit syncytia formation and cell–cell fusion induced by a several classes viral fusion proteins, and the degree of inhibition depends on the cell type [44]. In this study, we reported that IFITM3 also inhibited syncytium formation induced by PFV infection in HT1080 cells. IFITMs can inhibit the entry of a variety of viruses, most of which are viruses that enter cells in a pH-dependent manner. As a virus that enters cells in a pH-dependent manner [33], IFITM3 also inhibits PFV entry into host cells. Studies have shown that IFITM1 is primarily localized at the plasma membrane, whereas IFITM2 and IFITM3 are more localized to intracellular compartments and co-localize with lysosomal-associated membrane protein 1 (LAMP1), Rab7, or CD63 [9, 45]. Moreover, PFV Env-mediated fusion occurs at both the plasma membrane and in endosomes [46]. Therefore, although IFITM3 is more likely to inhibit PFV fusion in the endosomal membrane, the specific mechanism requires further exploration. Considering that IFITM1, IFITM2, and IFITM3 have a strong inhibitory effect on PFV replication, further investigation is warranted regarding whether IFITM1 can inhibit PFV fusion in the plasma membrane and whether IFITM2 inhibits PFV fusion in endosomes.

For FVs, envelope glycoprotein mediates viral entry into host cells and are also essential for viral budding [3]. The IFITM proteins were reported to inhibit the late step of feline foamy virus (FFV) replication without effect on early entry step, but the exact mechanism remains unclear [41]. In this study, we also found that IFITM3 downregulated the number of PFV envelope protein, and this reduction was associated with IFITM3 promoting envelope protein degradation via the lysosomes. These results suggest that, unlike FFV, IFITM3 inhibits both PFV entry into target cells and late step in the PFV life cycle. Previous studies have shown that IFITM3 can impair the processing of the HIV-1 envelope protein and degrades the envelope protein through lysosomes [42, 47]; however, the Ebola glycoprotein is insensitive to IFITM3 [42], suggesting that not all viral glycoproteins inhibited by IFITMs are affected by IFITMs.

Overall, these results suggest that in addition to inhibiting PFV entry, IFITM3 also reduces the abundance of envelope proteins that are essential for viral replication, and PFV can be inhibited via these two mechanisms.

## Conclusions

In conclusion, this study provides the first experimental evidence that IFITM1-3 can inhibit PFV, broadening the antiviral spectrum of IFITMs. We further demonstrate that IFITM3 could not only inhibit PFV entry into target cells but also reduce the abundance of PFV envelope protein, thereby providing insight into the comprehensive antiviral mechanism of IFITMs.

## Supplementary Information

**Additional file 1. Fig. S1**. IFITM3 knockdown in HT1080-shIFITM3 cells was specific. The mRNA levels of IFITM1, IFITM2 and IFITM3 in HT1080-shIFITM3 cells were detected by quantitative PCR. Values were statistically evaluated using a two-way ANOVA. Compared with the HT1080-shNC cells: *P < 0.05; **P < 0.01; ***P < 0.001; ****P < 0.0001. Error bars represent the standard deviations.

## Data Availability

All data generated or analyzed during this study are included in this published article.

## Abbreviations

FVs: Foamy viruses
PFV: Prototype foamy virus
IFITMs: Interferon induced transmembrane
IFN: Interferon
ISGs: Interferon stimulating genes
HIV: Including human immunodeficiency virus
EBOV: Ebola virus
IAV: Influenza A virus
ZIKV: Zika virus
SARS-CoV: Severe acute respiratory syndrome coronavirus
MLV: Murine leukemia virus
AAV: Adeno-associated virus
LCMV: Lymphocytic choroid plexus bacterial meningitis
LASV: Arenavirus
IAV: Including influenza A virus
CPE: Cytopathic effects
