# Supplementary material for: Antiviral role of IFITM3 in prototype foamy virus infection
Source: Virol J. 2022 Nov 22;19:195. doi: 10.1186/s12985-022-01931-x (PMC9682733; doi:10.1186/s12985-022-01931-x)
Supplement: Supplementary file 1 — Additional file 1. Fig. S1. IFITM3 knockdown in HT1080-shIFITM3 cells was specific. The mRNA levels of IFITM1, IFITM2 and IFITM3 in HT1080-shIFITM3 cells were detected by quantitative PCR. Values were statistically evaluated using a two-way ANOVA. Compared with the HT1080-shNC cells: *P < 0.05; **P < 0.01; ***P < 0.001; ****P < 0.0001. Error bars represent the standard deviations. [file 12985_2022_1931_MOESM1_ESM.docx]

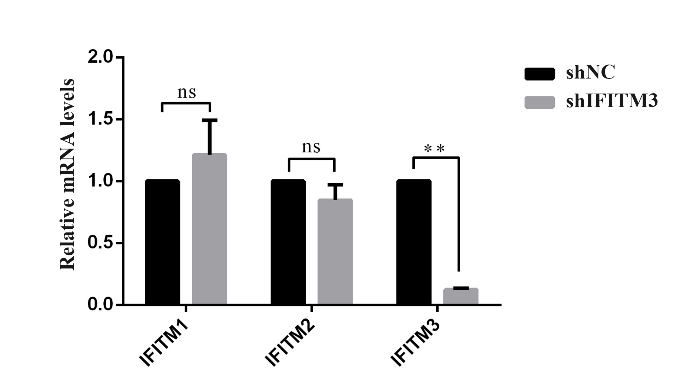


**Supplementary Figure S1. IFITM3 knockdown in** **HT1080-shIFITM3 cells was specific.** The mRNA levels of IFITM1, IFITM2 and IFITM3 in HT1080-shIFITM3 cells were detected by quantitative PCR. Values were statistically evaluated using a two-way ANOVA. Compared with the HT1080-shNC cells: *P < 0.05; **P < 0.01; ***P < 0.001; ****P < 0.0001. Error bars represent the standard deviations.
